# Identifying individuals with undiagnosed post-traumatic stress disorder in a large United States civilian population – a machine learning approach

**DOI:** 10.1186/s12888-022-04267-6

**Published:** 2022-09-29

**Authors:** Patrick Gagnon-Sanschagrin, Jeff Schein, Annette Urganus, Elizabeth Serra, Yawen Liang, Primrose Musingarimi, Martin Cloutier, Annie Guérin, Lori L. Davis

**Affiliations:** 1 Analysis Group, Inc., 1190 avenue des Canadiens-de-Montréal, 1190 avenue des Canadiens-de-Montréal, Tour Deloitte, Suite 1500, Montréal, QC H3B 0G7 Canada; 2 Otsuka Pharmaceutical Development & Commercialization, Inc., 508 Carnegie Center, Princeton, NJ 08540 USA; 3 Lundbeck LLC, 6 Parkway North, Deerfield, IL 60015 USA; 4 H. Lundbeck A/S, Ottiliavej 9, Valby, Copenhagen, Denmark; 5 Research Service, Tuscaloosa Veterans Affairs Medical Center, 3701 Loop Rd East, Tuscaloosa, AL 35404 USA; 6 Department of Psychiatry and Behavioral Neurobiology, University of Alabama Heersink School of Medicine, 1720 7th Avenue South, Birmingham, AL 35233 USA

**Keywords:** Civilian, Machine learning, Post-traumatic stress disorder, Undiagnosed

## Abstract

**Background:**

The proportion of patients with post-traumatic stress disorder (PTSD) that remain undiagnosed may be substantial. Without an accurate diagnosis, these patients may lack PTSD-targeted treatments and experience adverse health outcomes. This study used a machine learning approach to identify and describe civilian patients likely to have undiagnosed PTSD in the US commercial population.

**Methods:**

The IBM® MarketScan® Commercial Subset (10/01/2015–12/31/2018) was used. A random forest machine learning model was developed and trained to differentiate between patients with and without PTSD using non–trauma-based features. The model was applied to patients for whom PTSD status could not be confirmed to identify individuals likely and unlikely to have undiagnosed PTSD. Patient characteristics, symptoms and complications potentially related to PTSD, treatments received, healthcare costs, and healthcare resource utilization were described separately for patients with PTSD (Actual Positive PTSD cohort), patients likely to have PTSD (Likely PTSD cohort), and patients without PTSD (Without PTSD cohort).

**Results:**

A total of 44,342 patients were classified in the Actual Positive PTSD cohort, 5683 in the Likely PTSD cohort, and 2,074,471 in the Without PTSD cohort. While several symptoms/comorbidities were similar between the Actual Positive and Likely PTSD cohorts, others, including depression and anxiety disorders, suicidal thoughts/actions, and substance use, were more common in the Likely PTSD cohort, suggesting that certain symptoms may be exacerbated among those without a formal diagnosis. Mean per-patient-per-6-month healthcare costs were similar between the Actual Positive and Likely PTSD cohorts ($11,156 and $11,723) and were higher than those of the Without PTSD cohort ($3616); however, cost drivers differed between cohorts, with the Likely PTSD cohort experiencing more inpatient admissions and less outpatient visits than the Actual Positive PTSD cohort.

**Conclusions:**

These findings suggest that the lack of a PTSD diagnosis and targeted management of PTSD may result in a greater burden among undiagnosed patients and highlights the need for increased awareness of PTSD in clinical practice and among the civilian population.

**Supplementary Information:**

The online version contains supplementary material available at 10.1186/s12888-022-04267-6.

## Introduction

Post-traumatic stress disorder (PTSD) is characterized by the presence of four clusters of symptoms that may present after experiencing or witnessing a traumatic event [1, 2]. These symptom clusters are associated with the traumatic event and include intrusive and recurrent memories, avoidance of trauma-related stimuli, negative mood or cognitions, and marked arousal and reactivity [1, 2]. The psychosocial impact of PTSD on patients is substantial, with increased risk of suicide attempts [3, 4], disability and unemployment [5], and comorbid conditions such as depression and substance use disorder [6–8]. In addition, PTSD is associated with a substantial economic burden to society, with a recent study estimating the burden at $232.2 billion in the United States (US) [9].

The 1-year prevalence of PTSD is estimated at 2.6 to 6.0% in civilians and 6.7 to 11.7% in military populations, and is twice as common among women compared to men [10]. Historically, PTSD has been predominantly studied among military individuals, likely due to the high prevalence of PTSD in this population [11, 12]. However, this represents a minority (14%) of the overall PTSD population in the US, with 86% of the PTSD population comprising civilians [9]. In addition, recent issues like COVID-19 [13], civil unrest [14], and climate change [15] continue to occur around the globe, adding to the growing concern of increased exposure to natural and societal traumatic events among civilians. Thus, additional research in this already underrecognized and understudied population is more imperative than ever.

In both military and civilian populations, PTSD is known to be underdiagnosed [16], which may occur for multiple reasons, including patients being misdiagnosed with another mental health condition [6], patients not seeking help due to the stigma surrounding PTSD [16], patients’ lack of awareness of the condition/recognition of the symptoms [17], and patients’ lack of disclosure of traumatic history as this information is not routinely obtained by primary care physicians [18]. Indeed, studies have suggested that the proportion of patients with PTSD that remain undiagnosed may be substantial [6, 19–21], with one study reporting that only 11% of adult patients in primary care that met diagnostic criteria for PTSD had a recorded diagnosis of PTSD [6]. Without an accurate diagnosis, individuals may lack PTSD-targeted treatments, which may be associated with adverse outcomes like suicide attempts and overall poor quality of life, as well as a higher risk of sustained, long-term PTSD and depressive symptoms [16, 22, 23]. Accordingly, untreated individuals incur considerable care costs [23]. Notably, even among patients who are diagnosed with PTSD, many remain untreated [6, 16]. However, diagnosing PTSD is the first step towards proper and targeted management, since patients who receive a mental health diagnosis have more than 8-times higher odds of subsequently receiving mental health care [6].

Given the negative outcomes experienced by patients with untreated PTSD, undiagnosed PTSD is likely associated with a substantial clinical and economic burden as well. This suspected large burden warrants an improved method to identify these patients in real-world clinical practice, so that the impact of underdiagnosis and subsequently, undertreatment, on patients, their family, and society as a whole may be better understood. This is particularly important in the civilian population, where regular and systematic screening programs like those available for veterans are lacking [24].

Machine learning is an approach that has become increasingly used in the field of psychiatry in recent years to identify patients with a range of undiagnosed conditions from real-world, retrospective data sources [25–27]. Machine learning can be particularly useful when identifying undiagnosed patients with complex conditions such as PTSD, where a large number of characteristics and interactions must be considered and examined in the context of very heterogeneous populations and patient profiles [28]. Therefore, the current study implemented a machine learning approach to identify and describe commercially (i.e., privately) insured civilian adult patients likely to have undiagnosed PTSD in the US.

## Methods

### Data source

Data from the IBM® MarketScan® Commercial Subset (October 1, 2015 – December 31, 2018) were used. This database consists of employer- and health plan–sourced data containing medical and pharmacy claims data for beneficiaries, comprising employees, their spouses, and dependents who are covered by employer-sponsored private health insurance across all US census regions. The database includes records of inpatient (IP) services, IP admissions, outpatient (OP) services, prescription-drug claims, and other medical care. The database includes the employer-paid portion of payments and any out-of-pocket expenses incurred by patients. The database also includes standard demographic variables, such as age and gender; however, information on race is not available. Because Diagnostic and Statistical Manual of Mental Disorders, Fifth Edition (DSM-5) diagnoses are not available in claims data, International Classification of Diseases, Tenth Revision, Clinical Modification (ICD-10-CM) codes were used to identify symptoms and disorders based on clinical input.

Data are de-identified and comply with the requirements of the Health Insurance Portability and Accountability Act; therefore, no institutional review board approval was needed.

### Study design and sample selection

The analyses for this study were conducted based on a retrospective cohort design to identify three (3) groups: Actual Positive PTSD, Likely PTSD, and Without PTSD cohorts. The study population included civilian, commercially insured adults (aged 18–64 years) in the US. Patients with diagnosed PTSD (Actual Positive PTSD cohort) were identified as those with ≥2 PTSD diagnoses (ICD-10-CM: F43.1) on distinct dates and ≥ 2 psychiatric evaluations within a 3-month period beginning on or before the first observed PTSD diagnosis. Patients confirmed to not have PTSD (Actual Negative PTSD cohort) were identified as those without any diagnosis of reaction to severe stress/adjustment disorder (ICD-10-CM: F43) at any time and with evidence that a diagnosis of reaction to severe stress/adjustment disorder was ruled out based on the presence of ≥2 psychiatric evaluations within a 3-month period.

Patients likely or unlikely to have undiagnosed PTSD (Likely PTSD and Unlikely PTSD cohorts) were identified among patients for whom PTSD status could not be confirmed using available data (Unlabeled cohort). This cohort comprised patients without any diagnosis of reaction to severe stress/adjustment disorder and without evidence that a diagnosis of reaction to severe stress/adjustment disorder was ruled out. Patients likely or unlikely to have undiagnosed PTSD were defined based on model performance metrics, as described below. Patients unlikely to have undiagnosed PTSD and patients in the Actual Negative PTSD cohort comprised a representative sample of the general civilian population without PTSD (Without PTSD cohort); this population was used for descriptive comparison purposes only.

The index date was defined as the calendar date of the first observed PTSD diagnosis for patients in the Actual Positive PTSD cohort, the last calendar date followed by 6 months of continuous health plan enrollment for patients in the Actual Negative PTSD cohort, and a randomly selected calendar date within the most recent period of continuous health plan enrollment with at least 6 months of continuous health plan enrollment both before and after the index date for the Unlabeled cohort. For all three cohorts, the study period was defined as the 6-month period following the index date, until the earliest of the end of data availability (December 31, 2018), end of continuous health plan enrollment.

### Feature selection

Features were selected for inclusion in the machine learning model based on the scientific literature [1, 28–33], discussions with a clinical expert, and available medical history captured in claims data. Indicators of trauma were expected to be highly underreported in claims data given that traumatic events that occurred before the start of data availability or that were not associated with healthcare resource use could not be captured. Therefore, indicators of trauma were not used as a main feature in the model; instead, the model was constructed using information that is routinely collected in clinical practice. Features included information on patients’ demographic characteristics, clinical characteristics, symptoms and complications potentially related to PTSD, treatments received, and use of emergency department (ED) services. Features were identified among the Actual Positive PTSD and Actual Negative PTSD cohorts on the index date (demographic characteristics) or during the study period (clinical characteristics, symptoms and complications potentially related to PTSD, treatments received, and use of ED services).

Both binary variables (i.e., presence of the feature) and count variables (i.e., number of days a claim for the feature was observed) were included in the model. For example, whether or not a specific treatment was received was captured as a binary variable, while the number of prescription fills for the treatment was captured as a count variable [34]. In total, 490 features were available for modeling (Additional file 1).

### Statistical analysis

#### Random forest model development

A random forest machine learning model was developed and trained to differentiate between patients with and without PTSD using the Actual Positive PTSD and Actual Negative PTSD cohorts. A random forest model is a decision-tree based model, with each decision tree constructed based on a random sample of the data and a random selection of the features. This approach was chosen for its ability to model non-linear relationships between features and outcome variables and to accommodate large feature space [25]. The random forest model was implemented with a maximum of 200 trees, above which the model performance stabilized; default values of minimum node size (one), and depth of trees (indefinite) were selected. The most important features for the prediction of PTSD status were identified by the model; importance was measured by permutation (i.e., the amount of prediction error added to the model if a feature is lost).

The final random forest model, after feature reduction [35], was trained based on 324 predictive features and was then applied to the Unlabeled cohort to identify individuals likely and unlikely to have undiagnosed PTSD (Additional file 1).

#### Evaluation of model performance

The performance of the random forest model was assessed using measures of area under the ROC curve [AUC] and F-beta scores. The AUC provides an aggregate measure of the performance of the model across all classification thresholds. Generally, the higher the AUC, the better the model performance; a model that randomly predicts patients’ probability of having PTSD would have an AUC of 0.5, while a model that predicts patients’ probability of having PTSD with 100% accuracy would have an AUC of 1.0.

F-beta scores are a measure of model performance that consist of the weighted (harmonic) mean of model precision and model recall at each potential classification threshold. The value of beta indicates the relative weights placed on precision and recall, such that beta = 1 indicates precision and recall are weighted equally and beta < 1 indicates that precision is weighted more heavily than recall. Similar to the AUC, a higher F-beta score generally indicates better model performance. As this study did not aim to identify all undiagnosed patients with PTSD, but rather to be confident that patients predicted to have undiagnosed PTSD may indeed have PTSD, multiple beta values that weighted precision more heavily than recall were assessed.

#### Descriptive analysis of patient characteristics by PTSD status

Patient characteristics, including demographic and clinical characteristics, symptoms and complications potentially related to PTSD, treatments received, healthcare costs, and healthcare resource utilization (HRU) were described separately for the Actual Positive PTSD, Likely PTSD, and Without PTSD cohorts. Demographic characteristics (e.g., age, sex) were described on the index date, while clinical characteristics (e.g., Charlson Comorbidity Index [CCI], comorbidities) were reported during the study period. Symptoms and complications potentially related to PTSD were described during the study period and included those of general health or quality of life (e.g., sleep disturbances); behavioral symptoms or disorders (e.g., eating disorders); symptoms involving cognition or perception (e.g., somnolence, stupor); physiological symptoms or reactions (e.g., abnormal blood pressure, abnormal heart rate); substance use indicators (e.g., rehabilitation services); and mental, behavioral, and neurodevelopmental disorders (e.g., major depressive disorder [MDD], anxiety disorders), among others. Treatments received by patients in the three cohorts were described during the study period. All-cause healthcare costs (2018 USD) and HRU incurred during the study period comprised medical (IP, OP, and ED) and pharmacy components, and were reported per-patient-per-6-months (PPP6M). Means, standard deviations, and medians were described for continuous variables, and frequency counts and percentages for categorical variables. No statistical comparisons between cohorts were conducted; all differences reported in this study are numerical.

## Results

### Machine learning model performance

The AUC of the model was 0.75, which indicated that the current model could distinguish between patients with and without PTSD reasonably well [36]. The F-beta score was maximized at a classification threshold of 80% when precision was weighted 10-times more than recall. Based on this threshold, patients with a predicted probability of having PTSD of at least 80% were classified as likely PTSD.

### Identification of cohorts

A total of 2,124,496 patients were included in this study, of whom 44,342 (2.1%) were classified in the Actual Positive PTSD cohort, 35,021 (1.6%) were classified in the Actual Negative PTSD cohort, and 2,045,133 (96.3%) patients comprised the Unlabeled cohort. Among patients in the Unlabeled cohort, 5683 (0.3%) were identified by the machine learning algorithm as likely to have undiagnosed PTSD. The Without PTSD cohort included 2,074,471 patients in either the Actual Negative PTSD cohort or the Unlikely PTSD cohort. The top seven most important predictive features of PTSD identified by the machine learning algorithm were MDD, anxiety disorders, antiadrenergic medication use, bipolar disorder, musculoskeletal and connective tissue diseases, substance use/abuse, and physiological symptoms or reactions.

### Patient characteristics

Patient characteristics were similar among patients with diagnosed PTSD and those likely to have undiagnosed PTSD, with a mean age of 38.7 years in the Actual Positive PTSD cohort and 38.2 years in the Likely PTSD cohort (Table 1). Additionally, 73.5% of the Actual Positive PTSD cohort and 69.4% of the Likely PTSD cohort were female.

**Table 1:** Demographic and clinical characteristics

Number of patients	Actual Positive PTSD cohort		Likely PTSD cohort		Without PTSD cohort	
	***N*** = 44,342		***N*** = 5683		***N*** = 2,074,471	
**Demographic characteristics**						
**Age, years; mean ± SD [median]**	38.7 ± 13.0 [38.0]		38.2 ± 12.6 [38.0]		42.5 ± 13.1 [44.0]	
**Female, N (%)**	32,579	(73.5%)	3946	(69.4%)	1,464,405	(70.6%)
**Heath plan type, N (%)**						
Preferred provider organization plan	25,916	(58.4%)	3462	(60.9%)	1,203,878	(58.0%)
Health maintenance organization plan	5538	(12.5%)	656	(11.5%)	237,455	(11.4%)
Consumer-driven health plan	4463	(10.1%)	492	(8.7%)	193,161	(9.3%)
Non-capitated point-of-service plan	3229	(7.3%)	459	(8.1%)	180,090	(8.7%)
High deductible health plan	3003	(6.8%)	431	(7.6%)	184,075	(8.9%)
Comprehensive plan	1482	(3.3%)	99	(1.7%)	39,544	(1.9%)
Exclusive provider organization plan	315	(0.7%)	31	(0.5%)	15,225	(0.7%)
Capitated or partially capitated point-of-service plan	157	(0.4%)	19	(0.3%)	8399	(0.4%)
Unknown	239	(0.5%)	34	(0.6%)	12,644	(0.6%)
**Census region of residence, N (%)**						
South	16,210	(36.6%)	2284	(40.2%)	880,418	(42.4%)
Midwest/North Central	10,022	(22.6%)	1278	(22.5%)	420,312	(20.3%)
West	9151	(20.6%)	1232	(21.7%)	404,749	(19.5%)
Northeast	8872	(20.0%)	885	(15.6%)	367,195	(17.7%)
Unknown	87	(0.2%)	4	(0.1%)	1797	(0.1%)
**Clinical characteristics**						
**CCI score**^a^**, mean ± SD [median]**	0.3 ± 0.8 [0.0]		0.2 ± 0.6 [0.0]		0.2 ± 0.6 [0.0]	
**Most frequent diagnoses**^b^**, N (%)**						
General contact with health services	29,450	(66.4%)	3777	(66.5%)	1,100,899	(53.1%)
Musculoskeletal and connective tissue diseases	19,420	(43.8%)	2480	(43.6%)	552,880	(26.7%)
General symptoms and abnormal findings	18,758	(42.3%)	2434	(42.8%)	566,973	(27.3%)
Endocrine, nutritional, and metabolic diseases	16,256	(36.7%)	2075	(36.5%)	565,530	(27.3%)
Respiratory diseases	14,361	(32.4%)	1642	(28.9%)	435,604	(21.0%)
Genitourinary diseases	12,162	(27.4%)	1445	(25.4%)	369,407	(17.8%)
Digestive system diseases	9500	(21.4%)	1181	(20.8%)	250,069	(12.1%)
Skin and subcutaneous tissue diseases	8980	(20.3%)	864	(15.2%)	306,338	(14.8%)
Injury	8817	(19.9%)	1148	(20.2%)	195,100	(9.4%)
Nervous system diseases	7553	(17.0%)	924	(16.3%)	130,672	(6.3%)

*CCI* Charlson Comorbidity Index, *ICD-10-CM* International Classification of Diseases, Tenth Revision, Clinical Modification, *N* Number, *PTSD* Post-traumatic stress disorder, *SD* Standard deviation

^a^*Source*: Quan, H., Li, B., Couris, C. H., Fushimi, K., Graham, P., Hider, P., Januel, J. M., & Sundararajan, V. (2011). Updating and validating the Charlson Comorbidity Index and score for risk adjustment in hospital discharge abstracts using data from 6 countries. American Journal of Epidemiology, 173(6), 676–682

^b^Diagnoses were defined based on three-digit ICD-10-CM code categories. Diagnoses that may indicate a symptom or complication potentially related to PTSD have been excluded from this list and are reported in Fig. 2

**Fig. 1:**
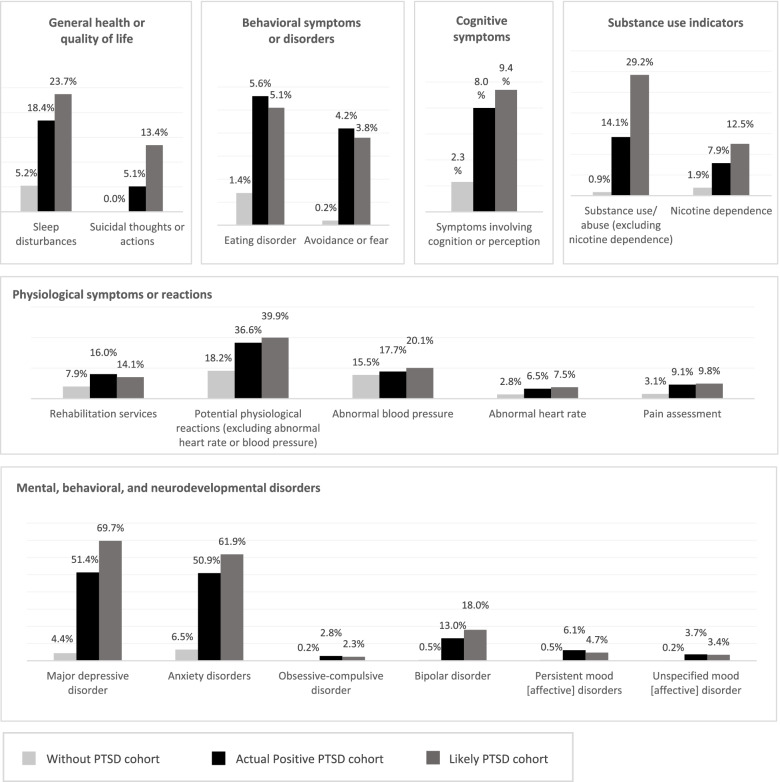
Symptoms and complications potentially related to PTSD^1^. ICD-10-CM: International Classification of Diseases, Tenth Revision, Clinical Modification; PTSD: post-traumatic stress disorder. Note [1] Symptoms and complications potentially related to PTSD were defined based on ICD-10-CM code categories

Several symptoms and complications potentially related to PTSD were also similar between patients with diagnosed PTSD and likely undiagnosed PTSD, including the frequency of eating disorders (5.6% in the Actual Positive PTSD cohort; 5.1% in the Likely PTSD cohort) and avoidance or fear (4.2% in the Actual Positive PTSD cohort; 3.8% in the Likely PTSD cohort; Fig. 1).

In contrast, other symptoms and complications were more common among patients likely to have undiagnosed PTSD than those with diagnosed PTSD, including sleep disturbances (18.4% in the Actual Positive PTSD cohort; 23.7% in the Likely PTSD cohort), suicidal thoughts or actions (5.1% in the Actual Positive PTSD cohort; 13.4% in the Likely PTSD cohort), and substance use (14.1% in the Actual Positive PTSD cohort; 29.2% in the Likely PTSD cohort), suggesting that certain symptoms may be exacerbated among those without a formal diagnosis.

Of note, comorbid mental health conditions were reported in over 50% of patients with diagnosed PTSD and over 60% of patients likely to have undiagnosed PTSD, with MDD (51.4% in the Actual Positive PTSD cohort; 69.7% in the Likely PTSD cohort) and anxiety disorders (50.9% in the Actual Positive PTSD cohort; 61.9% in the Likely PTSD cohort) among the most frequently observed mental health diagnoses. The frequency of these diagnoses among patients without PTSD was notably lower, with 4.4% of patients in the Without PTSD cohort having an observed diagnosis of MDD and 6.5% having an observed diagnosis of anxiety disorder.

### Treatments received

**Fig. 2:**
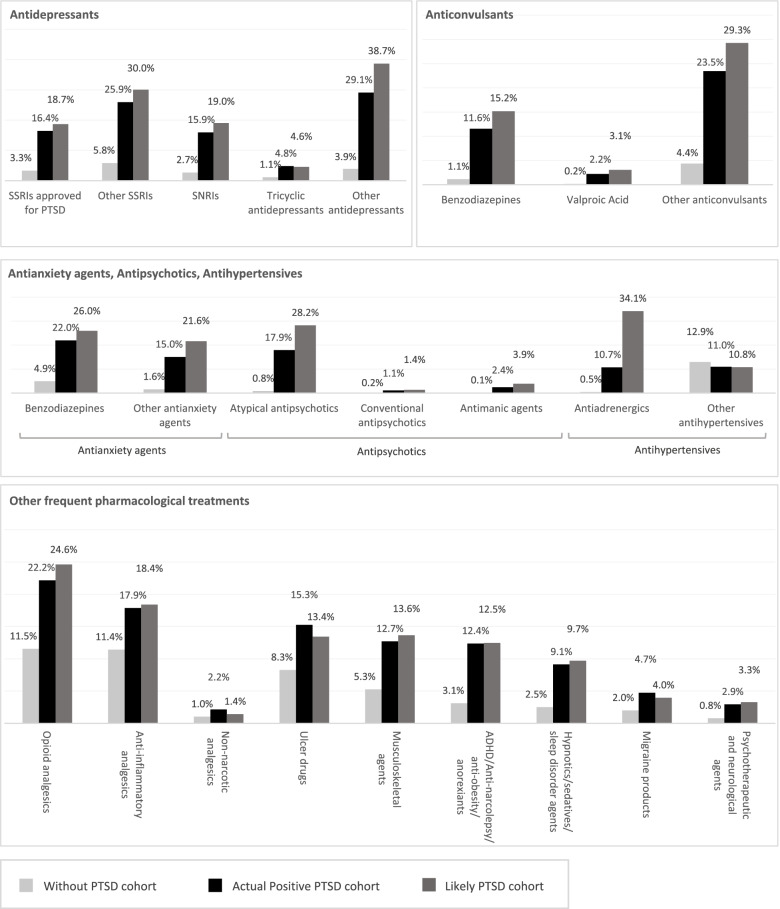
Treatments received^1^ ADHD: Attention Deficit Hyperactivity Disorder; GPI: Generic Product Identifier; PTSD: post-traumatic stress disorder; SNRIs: serotonin and norepinephrine reuptake inhibitors; SSRIs: selective serotonin reuptake inhibitors. Note [1] Treatments received were reported based on the GPI classification system and observed pharmacy claims

Use of certain treatments was similar among patients with diagnosed PTSD and those likely to have undiagnosed PTSD, including selective serotonin reuptake inhibitors (SSRIs) indicated for PTSD, antianxiety benzodiazepines, and anticonvulsant benzodiazepines (Fig. 2), which may suggest an overlap in the management of PTSD-related symptoms independent of diagnosis.

Conversely, the use of other treatments was more common among patients with likely undiagnosed PTSD than diagnosed PTSD, including atypical antipsychotics and antiadrenergics (Fig. 2).

### Healthcare costs and HRU

**Fig. 3:**
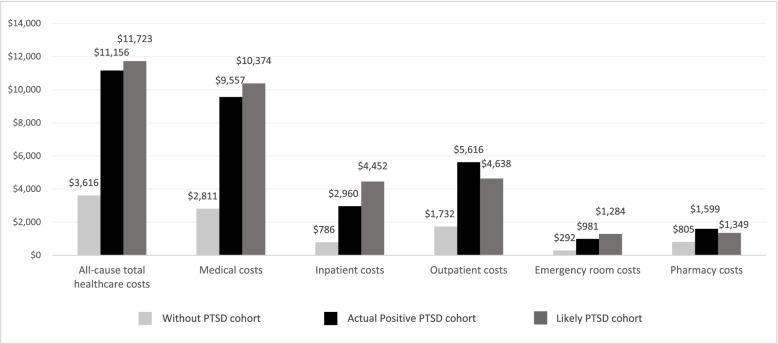
Mean healthcare costs (PPP6M; 2018 USD)^1^. PPP6M: per-patient-per-6-months; USD: United States dollar. Note [1] Healthcare costs were adjusted to 2018 USD using the US Medical Care Consumer Price Index, and were reported from a societal perspective (i.e., health plan payment and patients’ payment)

Mean all-cause total healthcare costs PPP6M were similar among patients with diagnosed PTSD ($11,156) and likely undiagnosed PTSD ($11,723), both of which were higher than the costs incurred by patients without PTSD ($3616). However, cost drivers differed between the two PTSD cohorts. Patients with likely undiagnosed PTSD incurred higher mean IP costs ($4452), but lower OP costs ($4638) than patients with diagnosed PTSD (IP: $2960; OP: $5616; Fig. 3).

Indeed, a higher proportion of patients with likely undiagnosed PTSD incurred ≥1 IP admission (22.5%) than those with diagnosed PTSD (12.0%), while patients with likely undiagnosed PTSD incurred fewer days with OP services (13.3 days) than those with diagnosed PTSD (18.3 days; Table 2).

**Table 2:** Healthcare resource utilization (PPP6M)

	Actual Positive PTSD cohort	Likely PTSD cohort	Without PTSD cohort
	***N*** = 44,342	***N*** = 5683	***N*** = 2,074,471
**Inpatient admissions, mean ± SD [median]**	0.2 ± 0.6 [0.0]	0.3 ± 0.7 [0.0]	0.0 ± 0.2 [0.0]
≥ 1 admission, N (%)	5305 (12.0%)	1278 (22.5%)	54,748 (2.6%)
**Inpatient days, mean ± SD [median]**	1.4 ± 6.2 [0.0]	2.4 ± 6.8 [0.0]	0.1 ± 1.5 [0.0]
**Days with emergency room services, mean ± SD [median]**	0.6 ± 1.6 [0.0]	0.7 ± 1.6 [0.0]	0.2 ± 0.7 [0.0]
**Days with outpatient services, mean ± SD [median]**	18.3 ± 14.2 [15.0]	13.3 ± 13.5 [10.0]	4.1 ± 6.4 [2.0]

*N* Number, *PPP6M* Per patient per 6 months, *PTSD* Post-traumatic stress disorder, *SD* Standard deviation

## Discussion

Identifying patients with undiagnosed PTSD is particularly challenging given the complexity of the condition and variability in symptom presentation and disease course, which may also overlap with other mental health conditions [18, 37]. Additionally, in the context of real-world studies, claims data lack available information regarding patients’ history of trauma, which is a defining feature of PTSD. Despite these challenges, the current study used novel machine learning techniques to identify patients likely to have undiagnosed PTSD by leveraging information routinely collected in real-world clinical settings, including recorded symptoms, diagnoses, and treatments. The top predictive features of PTSD that were identified, including MDD, anxiety disorders, substance use/abuse, and musculoskeletal and connective tissue diseases, have previously been associated with PTSD in the literature as common comorbidities [38, 39], thus enforcing the predictive capabilities of the machine learning algorithm.

Among patients identified by the machine learning algorithm, many similar characteristics were observed between patients likely to have undiagnosed PTSD and patients with diagnosed PTSD, including established symptoms, several mental health complications, and associated conditions and medications. Taken together, the model performance, similarities between patients likely to have undiagnosed PTSD and patients with diagnosed PTSD, and differences compared to those without PTSD, seem to indicate that the patients identified with likely undiagnosed PTSD indeed have PTSD.

Some important differences were also observed between patients likely to have undiagnosed PTSD and patients with diagnosed PTSD. For instance, the frequency of MDD and anxiety disorders was higher among patients likely to have undiagnosed PTSD, which may suggest potential misdiagnosis or missing diagnostic coding for concurrent PTSD. In line with these findings, an electronic medical record-based study of primary care clinics in the US by Meltzer et al. found that almost half of patients with PTSD were misdiagnosed as having depression [6]. In the current study, patients likely to have undiagnosed PTSD were also observed to have increased rates of PTSD complications, including sleep disturbances, suicidal thoughts, and substance use. This finding suggests that symptoms may be exacerbated among those without a formal PTSD diagnosis, potentially due to management being directed primarily toward addressing individual symptoms. However, further research is warranted to confirm these results, given that patients with undiagnosed PTSD were identified via a machine learning algorithm, and thus some patients may have been incorrectly classified as having PTSD. Taken together, these findings suggest that the lack of a PTSD diagnosis and targeted management of PTSD may result in a greater complication and comorbidity burden in undiagnosed patients. This hypothesis is aligned with studies that have shown poor quality of life and risk of sustained, long-term PTSD and depressive symptoms among individuals with untreated PTSD, which can lead to substantial healthcare costs [22, 23]. While healthcare costs measured in the current study were similar between patients with diagnosed PTSD and those with likely undiagnosed PTSD, the long-term impact of untreated PTSD could eventually result in much higher lifetime costs.

The results of this study highlight the need for increased awareness of PTSD in clinical practice and among the civilian population. Improved mental health literacy and recognition of PTSD as a serious mental health condition may help to reduce the stigma and negative perceptions around PTSD and related trauma. In turn, this may potentially reduce existing barriers to seeking appropriate mental health services, which may facilitate formal diagnosis among the currently undiagnosed civilian population. As a step towards this goal, the results of the current study could potentially contribute to future research regarding the development of a simple, accessible clinical screening tool that relies primarily on routinely collected information, rather than the disclosure of trauma history, to identify patients in the US civilian population that may benefit from formal PTSD diagnostic evaluation. Such a tool that does not rely on the disclosure of trauma history may have the potential to identify a broader range of patients that could benefit from PTSD screening, including those for whom sensitive information regarding trauma history is not routinely collected or readily disclosed in a primary care setting [18].

In the military population, systematic screening initiatives, such as the one established by the Department of Defense and Veterans Health Administration, have contributed to timely diagnoses, clinically relevant reduction of symptoms, and remission of PTSD among treated Army service members [40]. Additionally, improved methods of PTSD screening could facilitate access to PTSD-targeted management and support programs, which are currently limited in the civilian population. For instance, rehabilitation programs like Individual Placement and Support (IPS) have been successful in returning Veterans with disabling PTSD back to steady employment with better incomes, in addition to improving quality of life for the recovering individual [41, 42]. Access to similar screening and rehabilitation strategies to identify undiagnosed civilian individuals with PTSD in a timely manner and provide much-needed support may help to alleviate the burden of the condition on patients, stakeholders, and society as a whole.

### Limitations

The findings of this study should be considered in light of certain limitations. First, predictions made for the Unlabeled cohort could not be confirmed with the available data (i.e., it could not be clinically confirmed that patients predicted to have undiagnosed PTSD did in fact have PTSD). However, the classification threshold was selected to maximize model precision in order to increase confidence that patients included in the Likely PTSD cohort represented a subset of the civilian population with undiagnosed PTSD. Importantly, further studies using different machine learning models are warranted to confirm the identification algorithm and the variations observed between the different cohorts. Second, traumatic events were not used as a main feature in the model due to underreporting in claims data. Third, given the nature of claims data, it was not possible to identify all clinical characteristics (e.g., subjectively experienced symptoms) associated with PTSD. In these cases, proxies for such characteristics were identified through discussions with a clinical expert, as feasible. As such, it is possible that some clinical characteristics may have been missed or misclassified. Fourth, while a primary objective of this study was to identify patients likely to have undiagnosed PTSD, further studies are warranted to assess the incremental impact of undiagnosis on clinical and economic outcomes related to PTSD. Fifth, because patients included in the Actual Negative PTSD cohort had ≥2 psychiatric evaluations within a 3-month period and were not a random selection of individuals without PTSD, included patients were likely different from the general population (e.g., they may have had more comorbidities). Sixth, patients in the Actual Positive PTSD cohort were required to have ≥2 psychiatric evaluations within a 3-month period, which may have selected for patients with more medical service use (e.g., with more severe PTSD). Finally, claims databases are subject to coding errors and inaccurate or missing data, but nevertheless remain a valuable source of information on a large sample of patients in a real-world setting.

## Conclusion

These results highlight the importance of accurate and timely diagnosis of PTSD in order to potentially avoid complications associated with untreated PTSD that may result from non-targeted management of symptoms and lead to higher healthcare costs. Findings may have implications for the implementation of more accessible screening programs to aid in the identification of civilian patients that could benefit from early identification and treatment of PTSD.

## Supplementary Information

**Additional file 1.** Features used in the development of the random forest model. The “Full list” tab includes all 490 features that were available for modeling. This full list is broken down by category in the “Trauma indicators”, “Symptoms and complications”, “Other diagnoses”, and “Medications” tabs, which provide a description of how each predictor was defined. The “Importance scores” tab includes the 324 predictive features that were used to train the final random forest model (with the corresponding importance scores).

## Data Availability

The datasets generated and analyzed during the current study are not publicly available because they were used pursuant to a data use agreement. The data are available through requests made directly to IBM, via their website (https://www.ibm.com/products/marketscan-research-databases) or email (askibm@vnet.ibm.com).
